# High-quality permanent draft genome sequence of the *Parapiptadenia rigida*-nodulating *Cupriavidus* sp. strain UYPR2.512

**DOI:** 10.1186/1944-3277-10-13

**Published:** 2015-04-11

**Authors:** Sofie E De Meyer, Elena Fabiano, Rui Tian, Peter Van Berkum, Rekha Seshadri, TBK Reddy, Victor Markowitz, Natalia N Ivanova, Amrita Pati, Tanja Woyke, John Howieson, Nikos C Kyrpides, Wayne Reeve

**Affiliations:** 1 Centre for Rhizobium Studies, Murdoch University, Murdoch, Western Australia; 2 Instituto de Investigaciones Biológicas Clemente Estable, Montevideo, Uruguay; 3 Soybean Genomics and improvement laboratory Bldg 006, BARC-West USDA ARS 10300 Baltimore Blvd, Beltsville, MD 20705, USA; 4 DOE Joint Genome Institute, Walnut Creek, CA, USA; 5 Biological Data Management and Technology Center, Lawrence Berkeley National Laboratory, Berkeley, CA, USA; 6 Department of Biological Sciences, King Abdulaziz University, Jeddah, Saudi Arabia

**Keywords:** Root-nodule bacteria, Nitrogen fixation, Rhizobia, Betaproteobacteria, GEBA-RNB

## Abstract

*Cupriavidus* sp. strain UYPR2.512 is an aerobic, motile, Gram-negative, non-spore-forming rod that was isolated from a root nodule of *Parapiptadenia rigida* grown in soils from a native forest of Uruguay. Here we describe the features of *Cupriavidus* sp. strain UYPR2.512, together with sequence and annotation. The 7,858,949 bp high-quality permanent draft genome is arranged in 365 scaffolds of 369 contigs, contains 7,411 protein-coding genes and 76 RNA-only encoding genes, and is part of the GEBA-RNB project proposal.

## Introduction

Legumes establish symbiotic associations with a group of soil bacteria, rhizobia, able to fix atmospheric nitrogen (N_2_). Rhizobia elicit the formation of a symbiotic organ called a nodule comprising differentiated plant and bacterial cells. Differentiated rhizobia within nodules are termed bacteroids, and acquire the ability to fix nitrogen. Rhizobia are phylogenetically diverse including genera from the *Alphaproteobacteria* (*Allorhizobium*, *Azorhizobium*, *Bradyrhizobium*, *Ensifer*, *Mesorhizobium*, *Rhizobium*, etc.) as well as from the *Betaproteobacteria* (*Burkholderia*, *Cupriavidus*) [1,2].

The biological nitrogen fixation process significantly contributes to the development of sustainable agriculture reducing the use of supplies dependent on fuel and alleviating environmental impacts produced by the addition of chemical fertilizer [3]. Moreover, forestation with leguminous trees associated with rhizobia, “nitrogen-fixing trees”, has been successfully used for recovering degraded soils [4].

*Parapiptadenia rigida* (Benth.) Brenan, is a “nitrogen-fixing tree” belonging to the Piptadenia group from the Mimosoideae subfamily [5]. It is a multipurpose tree, very appreciated because of its timber and therefore used in high quality furniture and construction. It is also used for gums, tannins and essential oil extraction, has medicinal properties and is included in agroforestry and reforestation programs [4,6,7]. Taulé *et al.*[8] demonstrated that this species could be nodulated either by Alpha-rhizobia (*Rhizobium*) or by Beta-rhizobia (*Burkholderia* and *Cupriavidus*) with *Burkholderia* being the preferred natural symbiont of this legume. In the case of *Cupriavidus* sp. UYPR2.512, this strain was isolated from a nodule of a *P. rigida* plant grown in soils collected from Mandiyú native forest in Artigas, Uruguay. Isolated bacterial colonies of *Cupriavidus* sp. UYPR2.512 were able to nodulate and to promote the growth of *P. rigida,* as well as *Mimosa pudica* plants [8].

To our knowledge, the only published sequenced genome of a Beta-rhizobia belonging to the *Cupriavidus* genus so far is that of *C. taiwanensis* LMG 19424^T^[9]. Interestingly, the closest relative of *Cupriavidus* sp*.* UYPR2.512 is *C. necator* ATCC 43291^T^[8]. Here, we present the description of the *Cupriavidus* sp*.* UYPR2.512 high-quality permanent draft genome sequence and its annotation.

## Organism information

### Classification and features

**Figure 1:**
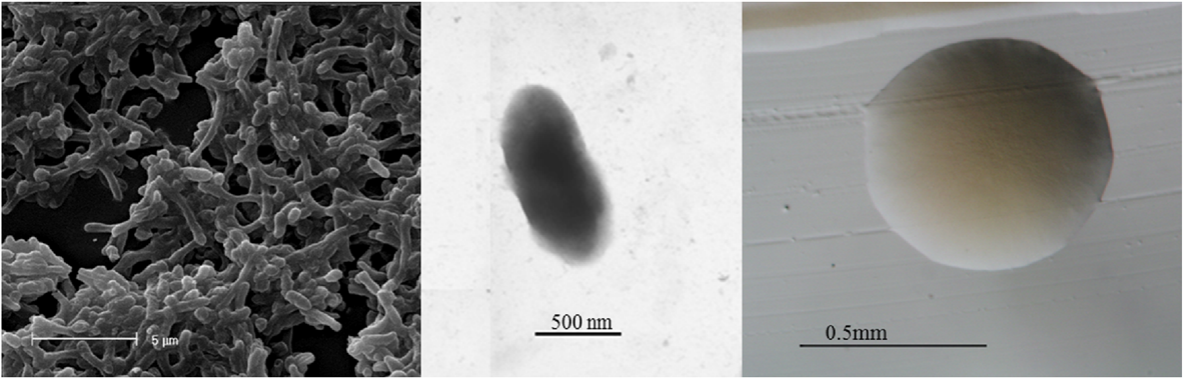
**Images of *Cupriavidus *** **sp. strain UYPR2.512 using scanning (Left) and transmission (Center) electron microscopy and the appearance of colony morphology on solid media (Right).**

*Cupriavidus* sp. strain UYPR2.512 is a motile, Gram-negative, non-spore-forming rod (Figure 1 Left, Center) in the order *Burkholderiales* of the class *Betaproteobacteria*. The rod-shaped form varies in size with dimensions of 0.5-0.7 μm in width and 0.9-1.2 μm in length (Figure 1 Left). It is fast growing, forming 0.5-0.8 mm diameter colonies after 24 h when grown on TY [10] at 28°C. Colonies on TY are white-opaque, slightly domed, moderately mucoid with smooth margins (Figure 1 Right).

**Figure 2:**
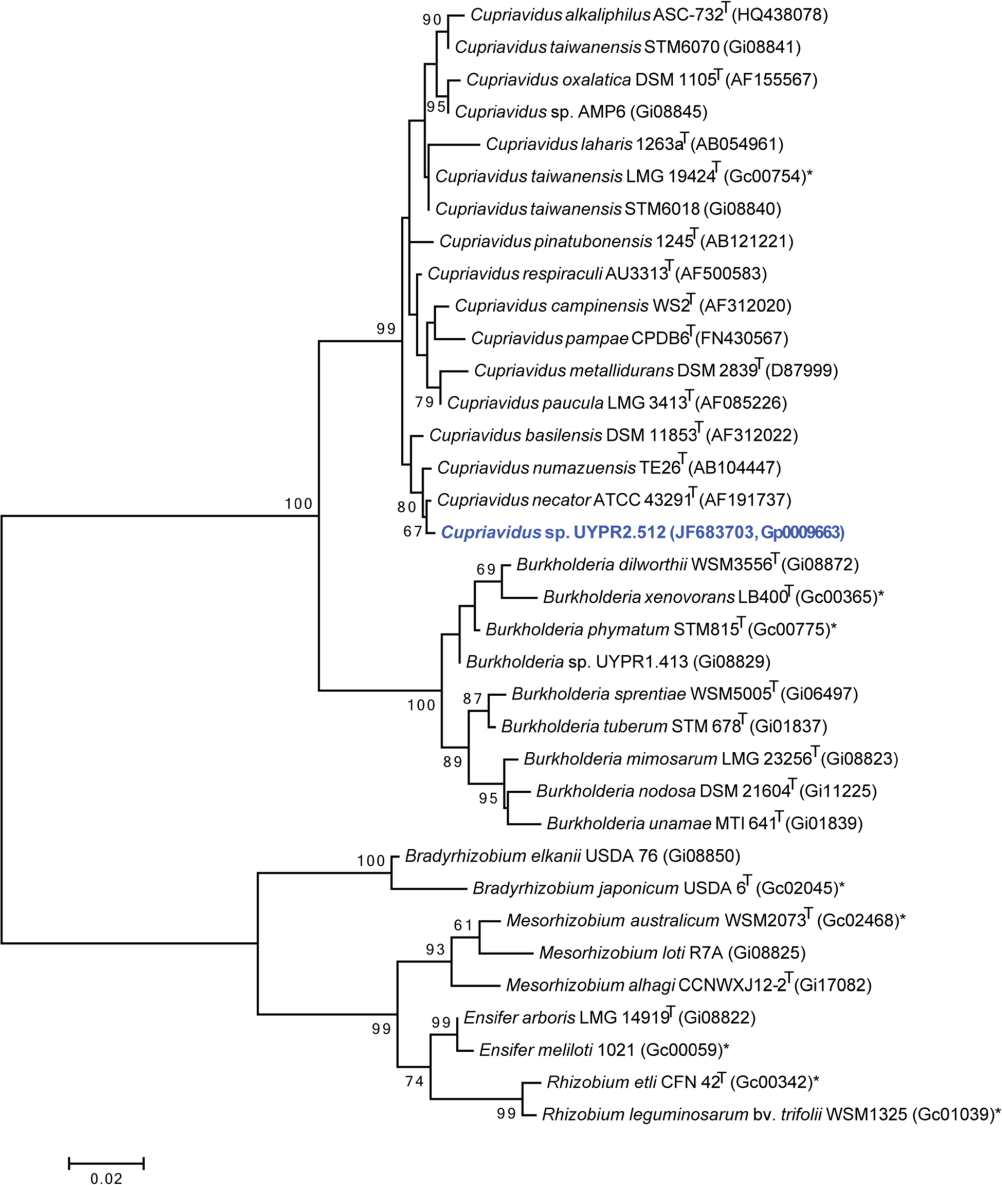
**Phylogenetic tree highlighting the position of *Cupriavidus *sp. strain UYPR2.512 (shown in blue print) relative to other type and non-type strains in the *Cupriavidus *genus using a 1,034 bp internal region of the 16S rRNA gene.** Several Alpha-rhizobia sequences were used as an outgroup. All sites were informative and there were no gap-containing sites. Phylogenetic analyses were performed using MEGA, version 5.05 [13]. The tree was built using the maximum likelihood method with the General Time Reversible model. Bootstrap analysis with 500 replicates was performed to assess the support of the clusters. Type strains are indicated with a superscript T. Strains with a genome sequencing project registered in GOLD [14] are shown in bold and have the GOLD ID mentioned after the strain number, otherwise the NCBI accession number has been provided. Finished genomes are designated with an asterisk.

Figure 2 shows the phylogenetic relationship of *Cupriavidus* sp. strain UYPR2.512 in a 16S rRNA gene sequence based tree. This strain is the most similar to *Cupriavidus necator* ATCC 43291^T^, *Cupriavidus oxalaticus* DSM 1105^T^ and *Cupriavidus taiwanensis* LMG 19424^T^ based on the 16S rRNA gene alignment with sequence identities of 99.32%, 98.49% and 98.42%, respectively, as determined using the EzTaxon-e server [11]. *Cupriavidus necator* ATCC 43291^T^ has been isolated from soil and is a non-obligate predator causing lysis of various Gram-positive and Gram-negative bacteria in the soil [12]. *Cupriavidus taiwanensis* LMG 19424^T^ is a plant symbiont and was isolated from root nodules of *Mimosa pudica* collected from three fields at Ping-Tung Country in the southern part of Taiwan [1]. Minimum Information about the Genome Sequence (MIGS) is provided in Table 1 and Additional file 1: Table S1.

**Table 1:** **Classification and general features of *Cupriavidus *** **sp. strain UYPR2.512 in accordance with the MIGS recommendations**[15]**published by the Genome Standards Consortium**[16]

**MIGS ID**	**Property**	**Term**	**Evidence code**
	Current classification	Domain *Bacteria*	TAS [17]
		Phylum *Proteobacteria*	TAS [18,19]
		Class *Betaproteobacteria*	TAS [20]
		Order *Burkholderiales*	TAS [21]
		Family *Burkholderiaceae*	TAS [22]
		Genus *Cupriavidus*	TAS [23]
		Species *Cupriavidus* sp.	IDA
	Gram stain	Negative	IDA [23]
	Cell shape	Rod	IDA
	Motility	Motile	IDA
	Sporulation	Non-sporulating	IDA [23]
	Temperature range	mesophile	IDA [23]
	Optimum temperature	28°C	IDA
	pH range; Optimum	Not reported	
	Carbon source	Not reported	
MIGS-6	Habitat	Soil, root nodule on host	IDA
MIGS-6.3	Salinity	Not reported	
MIGS-22	Oxygen requirement	aerobic	IDA
MIGS-15	Biotic relationship	Symbiotic	IDA
MIGS-14	Pathogenicity	Non-pathogenic	NAS
MIGS-4	Geographic location	Uruguay	IDA
MIGS-5	Nodule collection date	2006	IDA
MIGS-4.1	Latitude	-30.507	IDA
MIGS-4.2	Longitude	-57.71	IDA
MIGS-4.4	Altitude	58 m	IDA

Evidence codes – IDA: Inferred from Direct Assay; TAS: Traceable Author. Statement (i.e., a direct report exists in the literature); NAS: Non-traceable Author. Statement (i.e., not directly observed for the living, isolated sample, but based on a generally accepted property for the species, or anecdotal evidence). These evidence codes are from the Gene Ontology project [24].

### Symbiotaxonomy

*Cupriavidus* sp. strain UYPR2.512 was isolated from *Parapiptadenia rigida*, a Mimosoideae legume native to Uruguay [8]. This tree is native to South America, including south Brazil, Argentina, Paraguay, and Uruguay, and used by locals for timber and as a source of gums, tannins and essential oils [8]. *Cupriavidus* sp. strain UYPR2.512 is able to renodulate its original host and is highly efficient in fixing nitrogen with this host [8]. A selection of other host plants, including *Trifolium repens, Medicago sativa, Peltophorum dubium* and *Mimosa pudica* were tested for their ability to nodulate with UYPR2.512. Of these plants, strain UYPR2.512 was only able to nodulate and fix nitrogen effectively with *M. pudica*[8].

## Genome sequencing information

### Genome project history

This organism was selected for sequencing on the basis of its environmental and agricultural relevance to issues in global carbon cycling, alternative energy production, and biogeochemical importance, and is part of the Genomic Encyclopedia of Bacteria and *Archaea*, The Root Nodulating Bacteria chapter (GEBA-RNB) project at the U.S. Department of Energy, Joint Genome Institute [25]. The genome project is deposited in the Genomes OnLine Database [14] and the high-quality permanent draft genome sequence in IMG [26]. Sequencing, finishing and annotation were performed by the JGI using state of the art sequencing technology [27]. A summary of the project information is shown in Table 2.

**Table 2:** **Genome sequencing project information for *Cupriavidus *** **sp. strain UYPR2.512**

**MIGS ID**	**Property**	**Term**
MIGS-31	Finishing quality	Permanent-draft
MIGS-28	Libraries used	Illumina Std PE
MIGS-29	Sequencing platforms	Illumina HiSeq 2000
MIGS-31.2	Fold coverage	106.8 X Illumina
MIGS-30	Assemblers	Velvet 1.1.04, ALLPATHS-LG V.r41043
MIGS-32	Gene calling methods	Prodigal 1.4
	Locus Tag	A3A5
	Genbank ID	ARBE00000000
	Genbank Date of Release	September 16, 2013
	GOLD ID	Gp0009663 [28]
	BIOPROJECT	PRJNA165301
MIGS-13	Source Material Identifier	UYPR2.512
	Project relevance	Symbiotic N_2_fixation, agriculture

### Growth conditions and DNA isolation

*Cupriavidus* sp. strain UYPR2.512 was grown to mid logarithmic phase in TY rich media [10] on a gyratory shaker at 28°C. DNA was isolated from 60 mL of cells using a CTAB (Cetyl trimethyl ammonium bromide) bacterial genomic DNA isolation method [29].

### Genome sequencing and assembly

The draft genome of *Cupriavidus* sp. UYPR2.512 was generated at the DOE Joint Genome Institute [27]. An Illumina Std shotgun library was constructed and sequenced using the Illumina HiSeq 2000 platform which generated 29,312,424 reads totaling 4,396.9 Mbp [30]. All general aspects of library construction and sequencing performed at the JGI can be found at the JGI web site [31]. All raw Illumina sequence data was passed through DUK, a filtering program developed at JGI, which removes known Illumina sequencing and library preparation artifacts (Mingkun L, Copeland A, Han J. unpublished). Artifact filtered sequence data was then screened and trimmed according to the k–mers present in the dataset. High–depth k–mers, presumably derived from MDA amplification bias, cause problems in the assembly, especially if the k–mer depth varies in orders of magnitude for different regions of the genome. Reads with high k–mer coverage (>30x average k–mer depth) were normalized to an average depth of 30x. Reads with an average kmer depth of less than 2x were removed. Following steps were then performed for assembly: (1) normalized Illumina reads were assembled using Velvet version 1.1.04 [32] (2) 1–3 Kbp simulated paired end reads were created from Velvet contigs using wgsim [33] (3) normalized Illumina reads were assembled with simulated read pairs using Allpaths–LG (version r41043)[34]. Parameters for assembly steps were: 1) Velvet (velveth: 63 –shortPaired and velvetg: -very clean yes –exportFiltered yes –min contig lgth 500 –scaffolding no –cov cutoff 10) 2) wgsim (-e 0 –1 100 –2 100 –r 0 –R 0 –X 0) 3) Allpaths–LG (PrepareAllpathsInputs: PHRED 64 = 1 PLOIDY = 1 FRAG COVERAGE = 125 JUMP COVERAGE = 25 LONG JUMP COV = 50, RunAllpathsLG: THREADS = 8 RUN = std_shredpairs TARGETS = standard VAPI_WARN_ONLY = True OVERWRITE = True). The final draft assembly contained 369 contigs in 365 scaffolds. The total size of the genome is 7.9 Mbp and the final assembly is based on 839.6 Mbp of Illumina data, which provides an average of 106.8x coverage.

### Genome annotation

Genes were identified using Prodigal [35], as part of the DOE-JGI genome annotation pipeline [36,37] followed by a round of manual curation using GenePRIMP [38] for finished genomes and Draft genomes in fewer than 10 scaffolds. The predicted CDSs were translated and used to search the National Center for Biotechnology Information (NCBI) non-redundant database, UniProt, TIGRFam, Pfam, KEGG, COG, and InterPro databases. The tRNAScanSE tool [39] was used to find tRNA genes, whereas ribosomal RNA genes were found by searches against models of the ribosomal RNA genes built from SILVA [40]. Other non–coding RNAs such as the RNA components of the protein secretion complex and the RNase P were identified by searching the genome for the corresponding Rfam profiles using INFERNAL [41]. Additional gene prediction analysis and manual functional annotation was performed within the Integrated Microbial Genomes-Expert Review (IMG-ER) system [42] developed by the Joint Genome Institute, Walnut Creek, CA, USA.

## Genome properties

**Figure 3:**
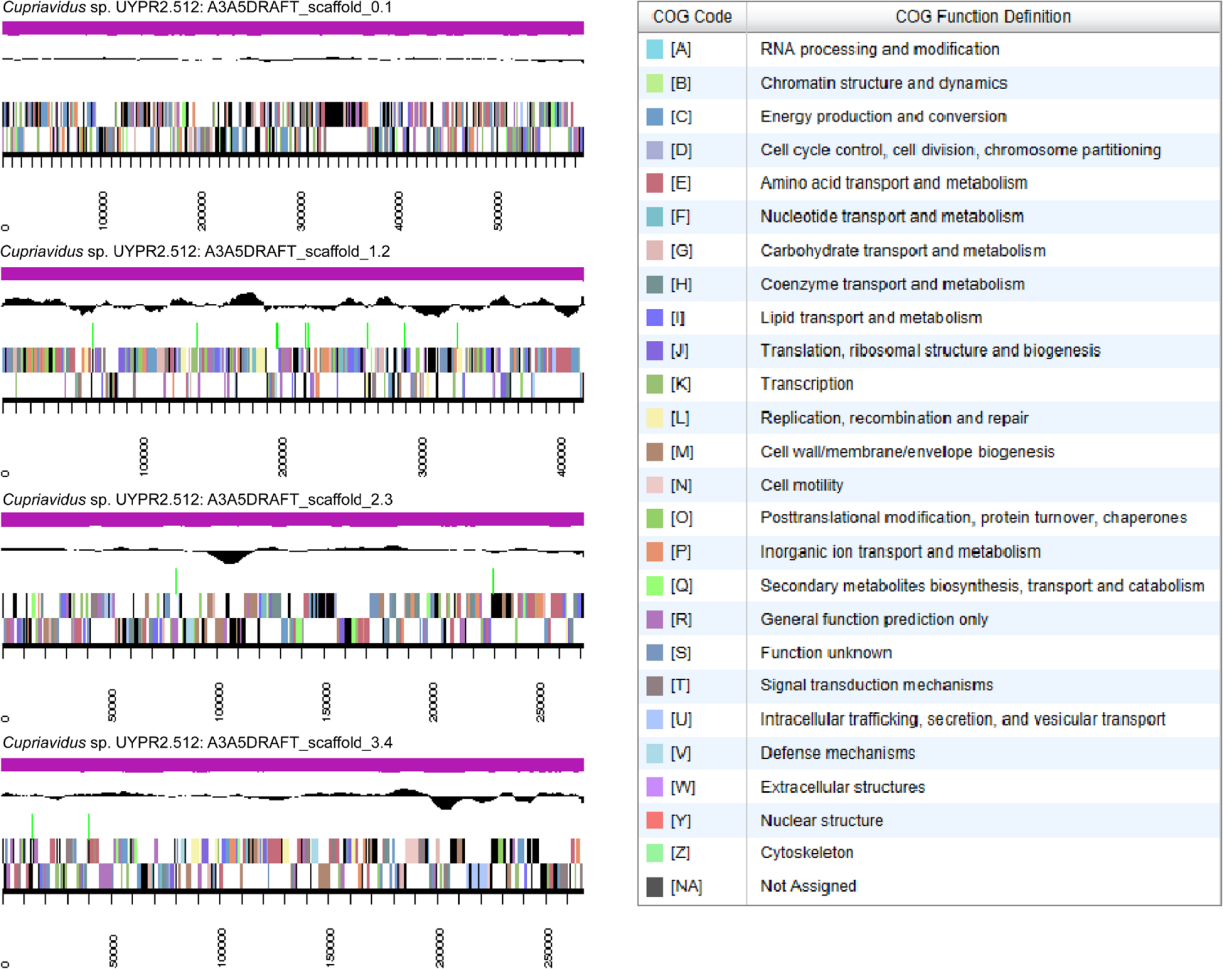
**Graphical map of the four largest scaffolds of the genome of *Cupriavidus *sp. strain UYPR2.512.** From the bottom to the top of each scaffold: Genes on forward strand (color by COG categories as denoted by the IMG platform), Genes on reverse strand (color by COG categories), RNA genes (tRNAs green, sRNAs red, other RNAs black), GC content, GC skew.

The genome is 7,858,949 nucleotides with 65.25% GC content (Table 3) and comprised of 365 scaffolds and 369 contigs (Figure 3). From a total of 7,487 genes, 7,411 were protein encoding and 76 RNA only encoding genes. The majority of genes (75.64%) were assigned a putative function whilst the remaining genes were annotated as hypothetical. The distribution of genes into COG functional categories is presented in Table 4.

**Table 3:** **Genome statistics for *Cupriavidus *** **sp. strain UYPR2.512**

**Attribute**	**Value**	**% of total**
Genome size (bp)	7,858,949	100
DNA coding (bp)	6,709,332	85.37
DNA G + C (bp)	5,128,158	65.25
DNA scaffolds	365	
Total genes	7,487	100
Protein coding genes	7,411	98.98
RNA genes	76	1.02
Pseudo genes	0	0
Genes in internal clusters	419	5.6
Genes with function prediction	5,663	75.64
Genes assigned to COGs	4,807	64.20
Genes with Pfam domains	5,959	79.59
Genes with signal peptides	696	9.30
Genes with transmembrane helices	1,545	20.64
CRISPR repeats	1	

**Table 4:** **Number of protein coding genes of *Cupriavidus *** **sp. strain UYPR2.512 associated with the general COG functional categories**

**Code**	**Value**	**% of total (5,426)**	**COG Category**
J	183	3.37	Translation, ribosomal structure and biogenesis
A	1	0.02	RNA processing and modification
K	526	9.69	Transcription
L	192	3.54	Replication, recombination and repair
B	3	0.06	Chromatin structure and dynamics
D	35	0.65	Cell cycle control, Cell division, chromosome partitioning
Y	0	0.00	Nuclear structure
V	56	1.03	Defense mechanisms
T	210	3.87	Signal transduction mechanisms
M	277	5.11	Cell wall/membrane/envelope biogenesis
N	99	1.82	Cell motility
Z	0	0.00	Cytoskeleton
W	0	0.00	Extracellular structures
U	140	2.58	Intracellular trafficking, secretion, and vesicular transport
O	181	3.34	Posttranslational modification, protein turnover, chaperones
C	434	8.00	Energy production and conversion
G	268	4.94	Carbohydrate transport and metabolism
E	487	8.98	Amino acid transport and metabolism
F	89	1.64	Nucleotide transport and metabolism
H	194	3.58	Coenzyme transport and metabolism
I	337	6.21	Lipid transport and metabolism
P	272	5.01	Inorganic ion transport and metabolism
Q	236	4.35	Secondary metabolite biosynthesis, transport and catabolism
R	661	12.18	General function prediction only
S	545	10.04	Function unknown
-	2680	35.80	Not in COGS

## Conclusion

*Cupriavidus* sp. UYPR2.512 belongs to a group of Beta-rhizobia isolated from *Parapiptadenia rigida,* a native tree from Uruguay belonging to the Mimosoideae legume group [8]. This tree is also native to the south of Brazil, Argentina and Paraguay [8]. Greenhouse experiments from previous studies have shown that *Cupriavidus* sp. UYPR2.512 is also able to nodulate and fix nitrogen with *Mimosa pudica*, an invasive species in many regions around the world [8]. Phylogenetic analysis revealed that UYPR2.512 is the most closely related to *Cupriavidus necator* ATCC 43291^T^, *Cupriavidus oxalaticus* DSM 1105^T^ and *Cupriavidus taiwanensis* LMG 19424^T^_._ In contrast to the other two strains, *Cupriavidus taiwanensis* LMG 19424^T^ is a microsymbiont that is able to nodulate and fix nitrogen in association with *Mimosa* species [43]. In total five *Cupriavidus* strains (AMP6, LMG 19424^T^, STM6018, STM6070 and UYPR2.512), which can form a symbiotic association have now been sequenced. A comparison of these strains reveals that UYPR2.512 has the largest genome (7.9 Mbp), with the highest KOG count (1398), the lowest G + C (65.25%) and signal peptide (9.3%) percentages in this group. All of these genomes share the nitrogenase-RXN MetaCyc pathway catalyzed by a multiprotein nitrogenase complex. Out of five *Cupriavidus* strains (AMP6, LMG 19424^T^, STM6018, STM6070 and UYPR2.512), which contain the N-fixation pathway, only *Cupriavidus* sp. UYPR2.512 has been shown to nodulate and fix effectively with *Parapiptadenia rigida*. The genome attributes of *Cupriavidus* sp. UYPR2.512 will therefore be important for ongoing molecular analysis of the plant microbe interactions required for the establishment of leguminous tree symbioses with this host.

## Competing interests

The authors declare that they have no competing interests.

## Authors’ contributions

EF supplied the strain and background information for this project, PVB supplied DNA to JGI, TR performed all imaging, SDM and WR drafted the paper, JH provided financial support and all other authors were involved in sequencing the genome and editing the final manuscript. All authors read and approved the final manuscript.

## Supplementary Material

Additional file 1 Associated MIGS Record. Click here for file
